# Biochemical assessment of metabolic associated fatty liver disease

**DOI:** 10.1515/almed-2021-0009

**Published:** 2021-03-15

**Authors:** Armando R. Guerra-Ruiz, Gregori Casals, Paula Iruzubieta, Marta Lalana, Alba Leis, Rosa María López, Javier Crespo, Manuel Morales-Ruiz

**Affiliations:** Commission on Biochemistry of Liver Disease, Spanish Society of Laboratory Medicine (SEQC-ML), Barcelona, Spain; Spanish Society of Digestive Pathology (SEPD), Madrid, Spain; Service of Clinical Analysis, Hospital Universitario Marqués de Valdecilla, Santander, Spain; Service of Biochemistry and Molecular Genetics, CDB, Hospital Clinic de Barcelona, IDIBAPS, CIBEREHD, Barcelona, Spain; Service of Digestive System, Hospital Universitario Marqués de Valdecilla, Clinical and Traslational Research Group on Digestive Disorders, IDIVAL, Santander, Spain; Service of Clinical Analysis, Hospital de Barbastro, Huesca, Spain; Service of Clinical Analysis and Biochemistry, Laboratori Clínic Metropolitana Nord, Hospital Universitari Germans Trias i Pujol, Badalona, Spain; Departments of Biochemistry and Microbiology, Unit of Liver Disease, Hospital Universitari Vall d’Hebron, Universitat Autònoma de Barcelona, Barcelona, Spain; Department of Biomedicine of the Faculty of Medicine and Health Sciences, Service of Biochemistry and Molecular Genetics, Hospital Clínic de Barcelona, IDIBAPS, CIBERehd, University of Barcelona, Barcelona, Spain

**Keywords:** hepatic steatosis, liver fibrosis, metabolic-associated fatty liver disease, serum markers, steatohepatitis

## Abstract

Metabolic-associated fatty liver disease (MAFLD) is defined as fat accumulation in the liver in the presence of metabolic alterations. This disorder is generally asymptomatic and may progress to severe liver disease, which are linked to inflammation and/or fibrosis. MAFLD has a high prevalence (26%) and therefore a considerable number of patients are at high risk of having advanced liver disease. This document provides an overview of the most relevant serological markers in the characterization and diagnosis of MAFLD. An example is provided of a routine diagnostic algorithm that incorporates serological testing. A range of useful serological scores are currently available for the management of MAFLD patients, especially for the stratification of patients at risk of fibrosis. A large proportion of the population is at risk of developing severe liver disease. The integration of non-invasive serological markers in the stratification of patients at risk for liver fibrosis may contribute to improve the control and management of MAFLD patients.

## Definition and introduction

Metabolic-associated fatty liver disease (MAFLD), previously known as non-alcoholic fatty liver disease (NAFLD) [1] encompasses a wide spectrum of liver lesions associated with the accumulation of fat in the liver, which is known as steatosis. In the past, diagnosis of NAFLD was only established after considerable alcohol consumption or drug-induced liver disease had been excluded. However, the new criteria for the diagnosis of MAFLD eliminate these exclusion criteria and incorporate “positive” criteria. Thus, diagnosis of MAFLD [2] is currently based on histological (biopsy), imaging, or serological evidence of liver fat accumulation (presence of steatosis), accompanied by one of the following criteria: overweight or obesity, type 2 diabetes mellitus, and/or evidence of metabolic disorders. The latter, which are also criteria for the diagnosis of metabolic syndrome, are described in Table 1.

**Table 1:** Evidence of metabolic alterations, defined as the presence of at least two of the following findings.

– Waist circumference ≥102 cm in men and ≥88 cm in women.
– Blood pressure ≥130/85 mmHg, or specific pharmacological treatment.
– Plasma triglycerides ≥150 mg/dL (≥1.70 mmol/L), or specific pharmacological treatment.
– Plasma HDL cholesterol <40 mg/dL (<1.0 mmol/L) for men and <50 mg/dL (<1.3 mmol/L) for women, or specific pharmacological treatment.
– Prediabetes (fasting glycemia: 100–125 mg/dL [5.6–6.9 mmol/L], or 2 h after glucose tolerance test: 140–199 mg/dL [7.8–11.0 mmol/L] or HbA1c: 5.7–6.4% [39 a 47 mmol/mol]).
– HOMA-IR (Homeostasis model assessment for assessing insulin resistance) ≥2.5.
– High-sensitivity C-reactive protein>2 mg/L.

MAFLD has become the leading cause of chronic liver disease in Western countries, with a prevalence of 24% in the general population [3]. MAFLD is strongly associated with the signs and symptoms of metabolic syndrome, with a higher prevalence among patients with this condition. The prevalence of steatohepatitis is 3–5% in the general population, which frequently has metabolic comorbidities such as diabetes and obesity [4]. About, 25% of these patients will develop cirrhosis and/or hepatocellular carcinoma (CHC).

In general terms, long-term mortality is higher in MAFLD patients as compared to the general population, with cardiovascular disease, cancer, and liver disease being leading causes of mortality, in that order. Considering the morbidity and mortality from cardiovascular and liver disease secondary to MAFLD, simple early diagnosis is essential to ensure the adequate management and treatment of these patients and reduce all-cause mortality.

**Table:** 


**The terms related to non-alcoholic fatty liver disease (NAFLD) must be replaced with those of metabolic-associated fatty liver disease (MAFLD), as this term better reflects current knowledge of the pathologic process and allows for a better categorization and management of patients.**

## Histopathology and natural history

MAFLD is categorized into two histological types: a) simple steatosis, which includes patients with hepatic steatosis with or without mild inflammation; and b) steatohepatitis, characterized by the presence of inflammation and hepatocyte injury (ballooning) with or without concomitant fibrosis [5], [6]. By histological analysis, fatty liver is characterized by: 1) simple steatosis, 2) steatosis with concomitant lobular or portal inflammation without ballooning, or 3) steatosis with concomitant ballooning but without inflammation. Diagnosis of steatohepatitis requires the presence of steatosis, ballooning, and lubular inflammation. Chronic liver inflammation may progress to fibrosis and eventually to cirrhosis.

**Figure 1:**
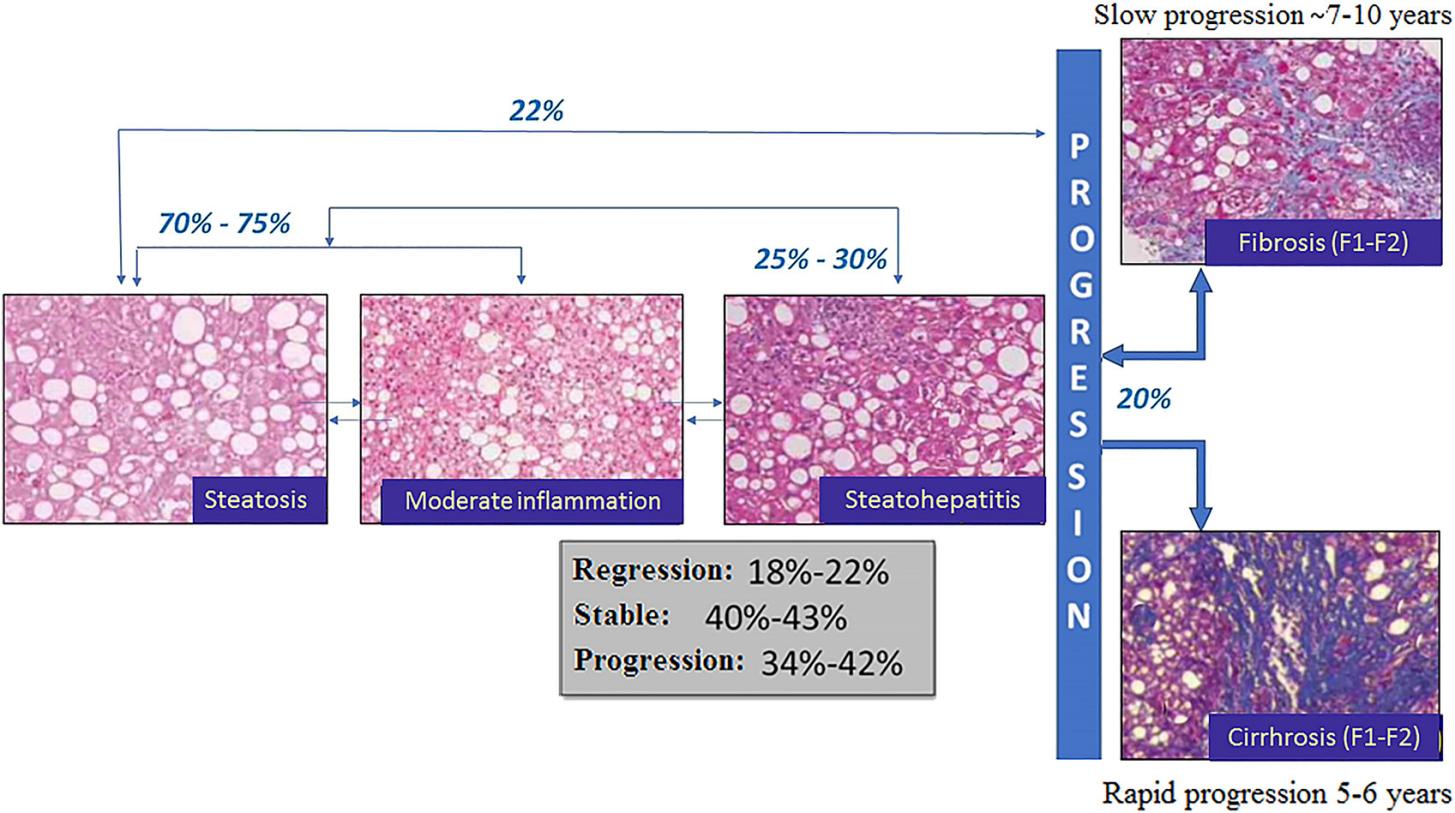
Natural history of metabolic associated fatty liver disease (MAFLD).

Figure 1 includes a diagram of the natural history of MAFLD. Although the mechanisms that induce the development and progression of MAFLD are still unclear, it is widely accepted that initial events are contingent on obesity and insulin resistance [7]. However, since not all MAFLD patients are insulin-resistant or obese, it is evident that both environmental and genetic factors play a role in the etiopathogenia of MAFLD [8]. Additionally, cases of fibrosis regression have been documented in patients with MAFLD, with reported percentages ranging from 15 to 33% according to the particularities of the group studied [9].

More specifically, 7% of cases steatohepatitis progresses to hepatocellular carcinoma (HCC) in a timeframe of 6.5 years. In 11% of cases, steatohepatitis progresses to cirrhosis in a period of 15 years [10]. Indeed, the presence of cirrhosis secondary to steatohepatitis in patients waiting for a liver transplant has tripled in the last decade, being the second leading cause of inclusion in the waiting list in the USA [11].

The main prognostic factor of MAFLD is the presence of liver fibrosis, since fibrosis determines the risk of cirrhosis, hepatic decompensation, and/or development of HCC. In other words, the presence of fibrosis determines the risk of mortality from liver disease. The degree of fibrosis is independently associated with all-cause mortality, including cardiovascular mortality, especially in the case of steatohepatitis [12], [13].

In MAFLD, the most widespread histological staging system for fibrosis is the one described by Brunt et al. [14], where S1 is defined as perisinusoidal or periportal fibrosis; S2 as perisinusoidal fibrosis with portal or periportal fibrosis; S3 as bridging fibrosis; and S4 as cirrhosis. The term “significant fibrosis” refers to S2 or a higher stage, and advanced fibrosis to S3 or a higher stage. Hepatic biopsy is the gold standard for the accurate diagnosis of MAFLD as well as for differential diagnosis of simple steatosis and steatohepatitis. This method categorizes the disease according to the level of activity (inflammation and cellular damage) and the stage of fibrosis. Biopsy, however, is an invasive method with an associated risk of complications besides some limitations, such as potential sampling errors and within- and inter-subject variability. For this reason, efforts are increasingly focused on the development of non-invasive diagnostic methods to detect steatohepatitis and fibrosis as first-line screening for identifying patients with severe liver disease and higher risk of mortality. Non-invasive methods include serological markers based on analytical parameters, as detailed below.

**Table:** 


**Determining the presence and degree of fibrosis in MAFLD is crucial, as this parameter is associated with all-cause mortality.**

## Analytical profile and diagnosis

Most patients with MAFLD remain asymptomatic, and suspicion of steatosis is raised by an incidental analytical finding that shows alterations in liver function markers. Another cause of suspicion is an alteration in liver morphology or echogenicity detected by an imaging study performed for another reason [15], [16].

MAFLD patients may exhibit elevated concentrations of aminotransferases, being MAFLD the main cause of persistent elevation of liver function markers. However, normal levels of aminotransferases do not exclude the presence of MAFLD. Indeed, most patients have normal aminotransferase concentrations [17], [18]. When aminotransferases are elevated, levels of alanine aminotransferase (ALT) and aspartate aminotransferase (AST) are slightly above the upper limit of normality. In the initial stages of steatosis, the AST/ALT ratio generally is<1. Reversal of this ratio may be suggestive of progression to fibrosis. Nevertheless, the degree of elevation of aminotransferases is not related to the degree of fibrosis or liver inflammation. As it occurs with aminotransferases, gamma-glutamyltransferase (GGT) is frequently elevated in MAFLD patients, and its elevation has been associated with a higher risk of fibrosis [19]. Alkaline phosphatase can also be slightly elevated, in parallel to other liver function markers.

Another frequent analytical finding is increased serum ferritin concentrations and transferrin saturation index. No evidence has yet been provided of a concurrent increase in hepatic iron deposition [20]. Likewise, patients may also frequently exhibit elevated serum autoantibody level, which is considered an epiphenomenon [21]. Some authors credit a prognostic value to this marker in liver disease [22], [23]. Bilirubin and albumin are rarely altered, except for patients with cirrhosis, who also exhibit prolonged prothrombin time, thrombocytopenia, and neutropenia.

On suspicion of MAFLD, ultrasonograpy is the first-line imaging study employed in routine practice because of its wide availability, low cost, and safety. The main limitation of this imaging study is that it has a limited sensitivity for the detection of mild steatosis. Thus, steatosis is not detected if it is<20% or in subjects with a BMI>40 [24]. In addition, ultrasonography is not useful for differentiating simple steatosis from steatohepatitis.

Differential diagnosis of simple steatosis vs. steatohepatitis along with staging of liver fibrosis are key factors in the diagnosis of MAFLD, as patients with steatohepatitis and fibrosis are at the highest risk of developing liver complications and cardiovascular disease. As it was mentioned above, biopsy is the gold standard for the histological staging of liver lesions. Nevertheless, we should not forget that liver biopsy is an invasive procedure with potential complications and its characteristics coupled with the high prevalence of fatty liver disease hinder the routine use of liver biopsy in a high proportion of patients. As a result, this technique is restricted to some clinical settings. Liver biopsy is only recommended to assess the presence of fibrosis, disease severity, co-occurrence of other liver diseases, and/or in patients at a higher risk of steatohepatitis and/or advanced fibrosis [5], [25], [26], who can be previously identified by using other less invasive techniques. This highlights the relevance of non-invasive methods in the diagnosis and staging of MAFLD, as they are useful in the characterization of patients and identification of high-risk patients [16], and spare low-risk patients from undergoing a liver biopsy.

Non-invasive methods are categorized into two groups: those based on “biological” or serum markers, and those based on “physical” or radiological markers. The latter include transient elastography (FibroScan^®^) and magnetic resonance elastography. Serum biochemical markers are gaining ground because of their validity, reproducibility, and easy use, emerging as a first-line technique in primary and secondary care for determining whether further studies are needed. Next, we will discuss the main serum markers identified to date for the detection of the three histological components of MAFLD (steatosis, inflammation, and fibrosis).

**Table:** 


**• The inclusion of GGT in basic liver panel may help in establishing the diagnosis and prognosis of MAFLD.**
**• In the management of MAFLD, non-invasive methods are useful to assess the probability that the patient has a severe histological lesion and requires a liver biopsy.**

## Steatosis scores

Several serum scores have been developed to predict the presence or not of hepatic steatosis: Fatty liver index (FLI) [27], SteatoTest [28], NAFLD-Liver Fat Score [29] and Hepatic Steatosis Index (HSI) [30]. Table 2 contains a summary of the variables that compose each index. These scores have been validated both, for the general population and population with obesity, are associated with insulin-resistance, and predict future metabolic, liver, and cardiovascular events with variable accuracy. These scores indicate the presence of steatosis with accuracy, although they do not allow for the quantification of liver fat [31].

**Table 2:** Predictive biochemical scores for hepatic steatosis.

	Biochemical markers	Other variables	Ref.
Fatty Liver Index (FLI)	Triglycerides, GGT	BMI, waist (cm)	[27]
Steatotest^a^	α2-macroglobulin, haptoglobin, apo A1, bilirubin, GGT, ALT, glucose, triglycerides, cholesterol	Age, sex, DM	[28]
NAFLD – Liver Fat Score	Insulin, AST, ALT	DM, metabolic syndrome	[29]
Hepatic Steatosis Index (HIS)	ALT, AST	BMI, DM	[30]

^a^Registered score. Apo A1, apolipoprotein A1; BMI, body mass index; DM, diabetes mellitus.

The most widespread and extensively validated tools are FLI and HSI. FLI values<30 exclude the presence of steatosis, with a likelihood ratio of 0.2; values≥60 are suggestive of steatosis, with a likelihood ratio of 4.3. An HSI<30 excludes MAFLD (with a negative likelihood ratio of up to 0.186), whereas an HSI≥36 confirms a diagnosis of MAFLD (with a minimum positive likelihood ratio of 6.069).

These scores may be influenced by the presence of liver inflammation and fibrosis and, as they do not offer advantages over routine imaging and analytical studies, their use is scarcely spread [16]. However, their wide availability, low cost and accessibility in primary care make them candidate to becoming a first-line screening tool for MAFLD. Some authors advocate FLI as first-line screening for advanced liver disease in the general population, as it they identify patients with steatosis [32].

**Table:** 


**• Because of their wide availability, serum scores should be the first-line screening test for steatosis in high-risk population.**
**• FLI and HSI emerge as the best option, as they have been widely validated in our population.**

## Serum markers of steatohepatitis

The serum markers investigated so far as potential markers of steatohepatitis are involved in the pathophysiological pathways of the disease (apoptosis/cell death, inflammation, and oxidative stress). The most extensively studied marker is cytokeratin-18 fragments (CK18-F), a product of hepatocyte apoptosis [33]. A meta-analysis reported an AUROC of 0.82 for CK18-F and a sensitivity and specificity of 75 and 77%, respectively, for the prediction of steatohepatitis, which indicates a very limited diagnostic accuracy. In addition, a wide variety of cut-off values were reported [34]. However, when this marker was incorporated to several panels, their diagnostic efficacy increased by up to 0.92 [35]. Further studies are needed to determine the utility of this marker.

Other markers studied include hormones such as the fibroblast growth factor 21 (FGF21) and adiponectin, with a very low diagnostic accuracy. Oxidative stress and inflammation markers such as interleukin 6 and tumor necrosis factor-*α* (TNF*α*) have also been studied. These markers have been assessed in small case series or pilot studies in heterogeneous groups of patients, with conflicting results. None of the markers studied so far could differentiate NASH from simple steatosis with a high sensitivity and specificity [36]. To improve the diagnostic accuracy of markers, several predictive models have been developed that combine some of these serum biomarkers with analytical parameters and clinical variables, including genetic polymorphisms. However, these models have not been adequately validated and are not recommended in clinical practice [37].

The evidence provided so far by metabolomic studies helped a Spanish research group to develop a test (OWL Liver Test) that differentiates steatohepatitis from simple steatosis, with good sensitivity and specificity (ROC>0.8). This test was developed using samples from a cohort of 465 patients [38] and has been validated in blind studies in two independent cohorts [39]. Limitations to the use of this test in other ethnic groups and patients with poor diabetes control were recently solved by the inclusion of transaminases and glycosylated hemoglobin (HbA1C) in the diagnostic algorithm [40]. The test is validated and marketed with the CE mark. The Spanish Society of Digestive Disorders (SEPD) recently launched a multicentric study, the *“NASH Registry”* aimed at assessing the usefulness of this tool.

**Table:** 


**Further studies are needed to validate the impact of serum markers of steatohepatitis in clinical practice.**

## Serum markers of liver fibrosis

Chronic liver disease is characterized by inflammation, ballooning, hepatocyte necrosis or apoptosis and fibrosis. However, fibrosis has been widely demonstrated to be the most determinant factor of liver disease progression [12], [41], [42], [43]. For this reason, staging the degree of fibrosis is mandatory in a patient with chronic liver disease. In addition, especially in the case of MAFLD, the degree of fibrosis has been found to be independently associated with cardiovascular risk and all-cause mortality [12], [13].

Diagnosis of cirrhosis is easy when the patient has already exhibited signs of decompensated liver disease (ascites, gastrointestinal bleeding due to esophageal varices, hepatic encephalopathy). However, it is in the early stages (S2 fibrosis or significant fibrosis) when prognosis can be changed the most. As patients with cirrhosis are generally asymptomatic, serum markers play a crucial role.

Serum markers of liver fibrosis are categorized into: 1) indirect markers of liver function, such as albumin, bilirubin, AST and ALT; and 2) direct markers, which are components of the extracellular matrix, including hyaluronic acid, matrix metalloproteinases, and collagen subtypes [44], [45]. A large proportion of these markers have been evaluated in patients with MAFLD, but none has shown a good diagnostic efficacy. The most effective approach is based on the use of predictive models that combine serum markers with the clinical characteristics of the patient [46], [47], [48], [49], [50], [51], [52], [53], [54], [55], [56], [57], [58], [59], [60], [61], [62], [63], [64], [65], [66], [67], [68], which are described in Table 3.

**Table 3:** Scores for non-invasive screening for liver fibrosis. Variables are categorized into direct and indirect biochemical markers and other variables.

	Indirect biochemical markers	Direct biochemical markers	Other variables	Ref.
NAFLD Fibrosis Score (NFS)	Glucose, platelets, AST, ALT, albumin	–	Age, BMI	[46], [47]
Fibrosis-4 (FIB-4)	Platelets, AST, ALT	–	Age	[58], [62]
Enhanced Liver Fibrosis (ELF)*		Hyaluronic acid, PIIINP, TIMP-1		[64], [65]
BARD score	AST, ALT		BMI, DM	[66]
AST to platelet ratio index (APRI)	AST, platelets			[48], [68]
FibroTest^a^	α2-macroglobulin, haptoglobin, apo A1, GGT, bilirubin		Age, sex	[49]
ActiTest	α2-macroglobulin, haptoglobin, apo A1, GGT, bilirubin			[50]
Hepascore^a^	Bilirubin, GGT, α2-macroglobulin	Hyaluronic acid	Age and sex	[51], [52]
PGA	Prothrombin index, GGT, apo A1			[53]
FibroIndex	Platelets, AST, Ύ-globulin			[54]
Forns	GGT, cholesterol, platelets		Age	[55]
Fibrometer NAFLD	Glucose, AST, ALT, ferritin, platelets		Age, weight	[48], [56]
FibroSpect II^a^	α2-macroglobulin	Hyaluronic acid, TIMP-1		[57]
SHASTA	AST, albumin	Hyaluronic acid		[59]
Hepamet Fibrosis Score (HFS)	AST, albumin, platelets, HOMA (glucose and insulin)		Age, sex, DM	[60]
ADAPT	Platelets	PRO-C3	Age, DM	[61]

^a^Registered scores. Apo A1, apolipoprotein A1; BMI, body mass index; DM, diabetes mellitus; PIIINP, amino-terminal propeptide of procollagen type III; TIMP-1, tissue inhibitor of metalloproteinases type 1.

There are two large groups of predictive models of advanced fibrosis: a) simple models, which use a combination of routine analytical parameters and clinical variables; and b) complex models, which use serum markers involved in the processing and degradation of the extracellular matrix (direct). It is important to note that the markers included in these predictive models of fibrosis can be influenced by other causes, such as thrombocytopenia unrelated to liver disease, elevated levels of GGT induced by antiretroviral therapies, alcohol consumption, or the use of medications with liver toxicity, Gilbert syndrome, hemolysis and cholestasis, to name a few [25], [56], [69], [70]. Direct markers better reflect extracellular matrix turnover as compared to matrix deposition. Concentrations of direct markers increase as the level of inflammation increases, whereas elevated matrix deposition can be underestimated when inflammation is minimal. However, none of these markers is specific to liver function and all can be influenced by other comorbidities and states (postoperative) associated with fibrosis. Therefore, concurrent extrahepatic inflammation can increase serum concentrations of a marker. Additionally, the release of some markers can be influenced by sinusoidal endothelial cell dysfunction or reduced bile excretion secondary to liver disease. Nevertheless, the accessibility, low cost and high negative predictive value of simple models make them a useful tool for initial diagnosis based on a critical interpretation of results. Table 4 shows the diagnostic value of high cut-off values for the main serological indices of advanced fibrosis in patients with MAFLD.

**Table 4:** Diagnostic value of high cut-off values for the main serological scores of advanced fibrosis in patients with MAFLD.

	Cut-off	AUROC	Sens, %	Spec, %	PPV, %	PNV, %	Ref
NFS	≥0.676	0.84	43	96	82	80	[66]
ELF	>9.8	0.90	80	90	71	94	[65]
FIB-4	≥3.25	0.80	26	98	75	85	[63]
APRI	≥1	0.77	43	86	34	90	[67]
HFS	≥0.47	0.87	35	97	72	82	[60]

AUROC, area under the ROC curve; Sens, sensitivity; Spec, specificity; PPV, positive predictive value; NPV, negative predictive value; NFS, NAFLD Fibrosis Score; ELF, Enhanced Liver Fibrosis; HFS, Hepamet Fibrosis Score.

The most extensively studied predictive models of fibrosis that have been validated for different populations of patients with MAFLD are Fibrosis-4 (FIB-4) and NAFLD Fibrosis Score (NFS). FIB-4 values<1.30 help exclude the presence of advanced fibrosis with a NPV of 95%; whereas values>3.25 indicate advanced fibrosis with a PPV of 75% [63]. With regard to NFS, a multicentric study in more than 700 patients reported a NPV of 88–93% to exclude advanced fibrosis for values <−1,455; and a PPV of 82–90% for values>0.676 [71]. The influence of comorbidities such as advanced age, diabetes and obesity on the results of these scores should always be considered. Another index, the Hepamet fibrosis score (HFS) was recently developed and validated in Spain. HFS has shown a significantly higher diagnostic Odds Ratio for the low (<0.12) and high (>0.47) cut-off values than FIB-4 and NFS to exclude/diagnose advanced fibrosis irrespective of the age, BMI, and the presence of diabetes [60]. Thus, HFS emerges as the most reliable indirect marker of liver fibrosis at this moment.

ADAPT is another promising score that was recently published, which formula includes age, the presence of diabetes, platelet count, and PRO-C3. PRO-C3 is a marker of collagen III formation that has not only been documented to be involved in the etiology of MAFLD, but also to increase with fibrosis [61]. The reported AUROC for this score is 0.89 both, for the derivation and the validation cohort, which exceeds the diagnostic accuracy of FIB-4 and NFS.

**Table:** 


**• Serum marker scores of fibrosis are a first screening approach for advanced fibrosis in patients with MAFLD.**
**• Both, FIB-4 and NFS have been validated for MAFLD and show a high diagnostic performance. The use of HFS is also recommended.**
**• When serum marker scores are suggestive of advanced fibrosis, further biochemical, radiological, and/or biopsy studies should be performed.**
**• During the critical evaluation of the scores obtained, the potential influence of other factors unrelated to metabolic liver disease must be taken into account.**

**Figure 2:**
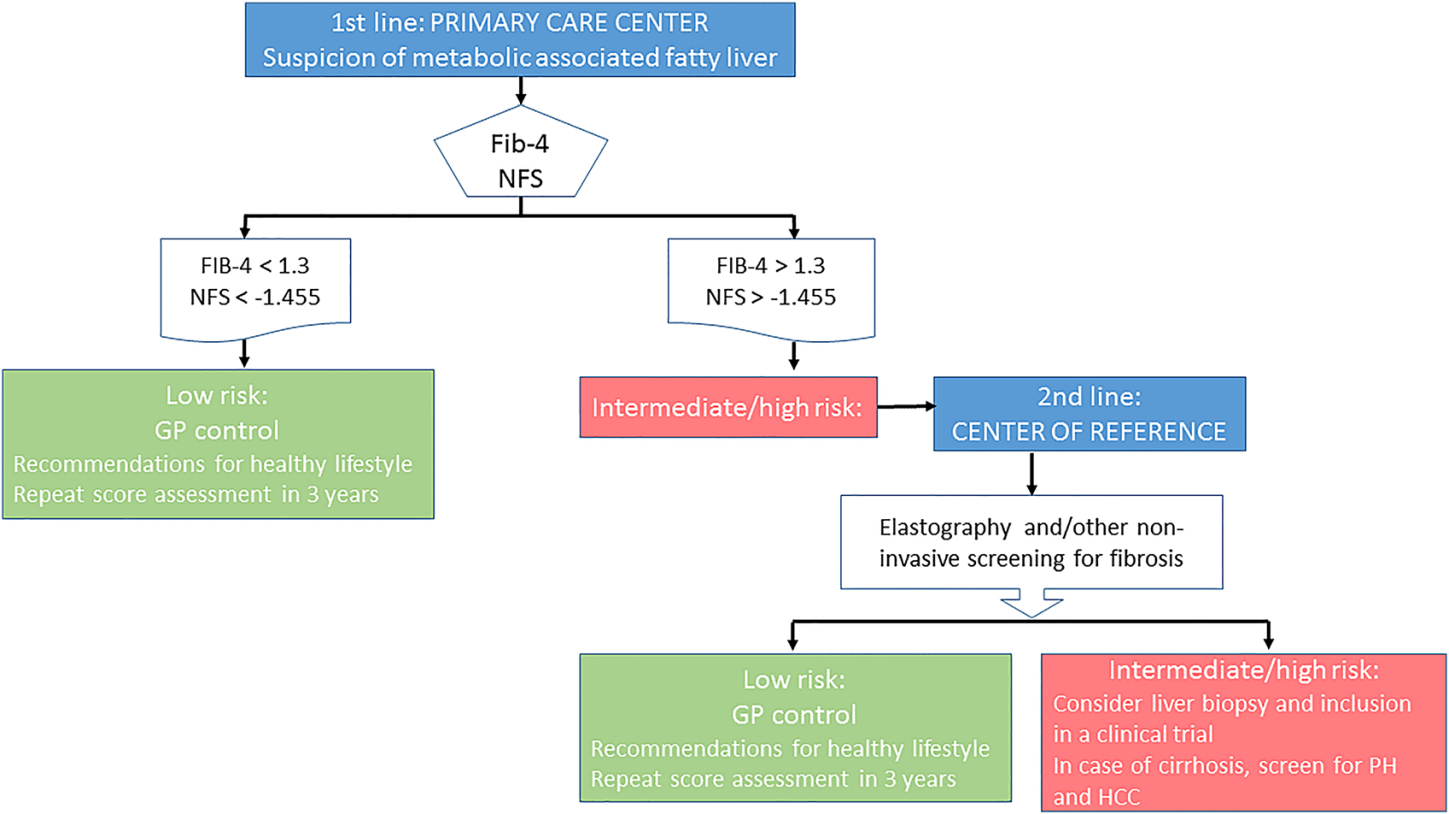
Proposal for an algorithm for the detection of liver fibrosis based on risk stratification using non-invasive markers in patients with suspected metabolic fatty liver. NFS, NAFLD Fibrosis Score; PH, portal hypertension; HCC, hepatocellular carcinoma.

## Use in clinical practice

On suspicion of MAFLD, non-invasive risk assessment should be performed in routine practice. The choice will depend on the local availability of the test. In Primary Care, the simplicity, low cost and wide availability of simple serum scores of fibrosis with a high negative predictive value make them the best first-line screening test.

As mentioned above, serum markers have some limitations or may be affected by a number of confounding factors that should be taken into account in the interpretation of results.

With regard to radiological markers, acoustic radiation force impulse (ARFI), Shear Wave elastography (SWE), and transient elastography with measurement of controlled attenuation parameter (CAP) can assess steatosis and fibrosis simultaneously [72]. The limitations of these techniques are similar to those of serum markers. The efficacy of FibroScan^®^ is limited by the presence of morbid obesity or a narrow intercostal space. Results can be influenced by any process that alters liver tenderness such as steatosis, venous congestion induced by heart failure, or acute inflammation secondary to acute hepatitis. Limitations of MRI-based methods include their limited availability and high cost. For this reason, some authors propose the combination of serum markers and radiological markers, being the most widely used FibroScan [73], [74], [75]. This combination increases the diagnostic accuracy of advanced fibrosis and reduces the need to perform a liver biopsy significantly. However, the studies included should not be performed simultaneously, as, although this combination provides a high sensitivity and specificity, the area of uncertainty increases [75]. In contrast, when these studies are performed sequentially, the area of uncertainty decreases while keeping a fine sensitivity and specificity [76].

The development of sequential diagnostic algorithms of MAFLD should start with widely-validated, low-cost serum scores with a higher negative predictive value such as FIB-4 or NFS. Patients with FIB-4<1.3 or NFS<−1.455 are considered to be at a low risk of having advanced fibrosis and further diagnostic testing is not required. However, patients at an intermediate (FIB-4 between 1.45 and 3.25 or NFS from −1.455 to 0.675) or high risk for advanced fibrosis (FIB-4>3.25 or NFS>0.675) should be referred to a center of reference for further testing of liver function with radiological or direct serum markers. For diagnosis of advanced fibrosis in patients with steatohepatitis, the combination of FIB-4 or NFS with ELF is recommended by the NICE (The National Institute of Health, UK) [77].

Figure 2 proposes an approach to MAFLD based on the clinical evidence currently available. This model initiates with a suspicion of MAFLD roused by the presence of steatosis detected by ultrasound, or the presence of metabolic risk factors (obesity, type 2 diabetes mellitus or metabolic syndrome) [37, 78].

**Table:** 


**• On suspicion of MAFLD, non-invasive risk assessment studies should be performed in routine practice.**
**• The development of sequential diagnostic algorithms of MAFLD should start with widely-validated, low-cost serum scores with a higher negative predictive value such as FIB-4 or NFS.**
**• In patients with serum marker scores not suggestive of significant fibrosis do not need further testing and can be reevaluated with a new measurement of serum marker scores in a year.**

## An opportunity for early diagnosis of liver disease

Clinical laboratories have a golden opportunity to contribute to the control, diagnosis and staging of the most frequent liver disease, metabolic-associated fatty liver disease. This opportunity involves incorporating above mentioned scores of steatosis and fibrosis, which can be estimated automatically, thereby contributing to a better awareness of the disease. This practice should not only be promoted by scientific societies of Laboratory Medicine, but also of Family and Community Medicine, Internal Medicine, Endocrinology, among others.

## Conclusions

MAFLD has become the first cause of chronic liver disease in Western countries, as it is closely related to obesity and metabolic syndrome. The main factors that determine progression of liver disease are inflammation and, especially, fibrosis. Fibrosis in MAFLD patients has not only been associated with liver-disease morbidity and mortality, but also with cardiovascular risk. This fact highlights the relevance of staging fibrosis in all patients diagnosed with MAFLD. In this context, serum markers are useful for the non-invasive screening of patients at a potential risk of advanced liver disease.
